# Associations between isolated and combined olfactory dysfunction and cognitive impairment with future dementia in community‐dwelling older adults

**DOI:** 10.1002/alz70857_105482

**Published:** 2025-12-25

**Authors:** Javier Oltra, Ingrid Ekström, Maria Larsson, Jane Yan, Giulia Grande, Erika J Laukka

**Affiliations:** ^1^ Aging Research Center, Department of Neurobiology, Care Sciences and Society, Karolinska Institutet and Stockholm University, Stockholm, Stockholm, Sweden; ^2^ Gösta Ekman Laboratory, Stockholm University, Stockholm, Sweden; ^3^ Stockholm Gerontology Research Center, Stockholm, Stockholm, Sweden

## Abstract

**Background:**

The combination of olfactory and cognitive markers has shown promising results in classifying individuals at different risk levels of dementia. We aimed to examine the association of isolated and combined olfactory dysfunction (OD) and cognitive impairment (CI) with incident dementia across 12 years and across two timeframes (0‐6 years and 6‐12 years) to evaluate whether the association varies over time.

**Method:**

The sample was comprised of 2406 older adults (*M*_age_=71.5 years, %_females_=60.8%) from the Swedish National Study on Aging and Care in Kungsholmen (SNAC‐K), free of dementia at baseline with available baseline odor identification (Sniffin’ Sticks test, range 0–16) and cognition (five cognitive domains) data. Participants were classified as having OD (performance <11) and as CI no dementia (CIND, **≥**1.5 *SD* below the age‐specific mean in at least one domain). CIND was further classified based on memory impairment (amnestic vs. non‐amnestic). Dementia hazard was estimated with Cox regression analyses for the whole study period (baseline to 12‐year follow‐up) and two timeframes (baseline to 6‐year follow‐up and 6‐ to 12‐year follow‐up) for isolated OD, isolated CIND, and their combination. Laplace regression was applied to assess the time until 5% of participants in each group received a dementia diagnosis, based on the 5% of incident dementia in the unimpaired participants (reference) over the whole period.

**Result:**

There were 1403 unimpaired, 326 isolated CIND, 476 isolated OD, and 203 CIND+OD individuals. CIND+OD was associated with increased dementia risk over the first 6‐year follow‐up (HR, 95% CI: 11.38, 6.70–19.32), more pronounced for amnestic CIND (22.23, 11.79–41.90). Isolated OD was associated with increased dementia risk over both periods (baseline to 6‐year follow‐up: 2.56, 1.48–4.43, baseline to 12‐year follow‐up: 2.12, 1.41–3.19), while isolated CIND was associated with dementia only over the first period (3.38, 1.75–6.49). 5% of CIND+OD individuals progressed to dementia 5 years after baseline, while isolated CIND and isolated OD reached the same proportion after 8 years.

**Conclusion:**

Concurrent CI and OD may signal incipient dementia in the coming years, especially for amnestic individuals. OD is a potential early marker of dementia on its own.

